# Estimating risk of consequences following hypoglycaemia exposure using the Hypo-RESOLVE cohort: a secondary analysis of pooled data from insulin clinical trials

**DOI:** 10.1007/s00125-024-06225-1

**Published:** 2024-07-22

**Authors:** Joseph Mellor, Dmitry Kuznetsov, Simon Heller, Mari-Anne Gall, Myriam Rosilio, Stephanie A. Amiel, Mark Ibberson, Stuart McGurnaghan, Luke Blackbourn, William Berthon, Adel Salem, Yongming Qu, Rory J. McCrimmon, Bastiaan E. de Galan, Ulrik Pedersen-Bjergaard, Joanna Leaviss, Paul M. McKeigue, Helen M. Colhoun

**Affiliations:** 1 Usher Institute, College of Medicine and Veterinary Medicine, University of Edinburgh, Edinburgh, UK; 2 Swiss Institute of Bioinformatics, Lausanne, Switzerland; 3 Division of Clinical Medicine, University of Sheffield, Sheffield, UK; 4 Medical & Science, Insulin, Clinical Drug Development, Novo Nordisk A/S, Soeberg, Denmark; 5 Diabetes Medical Unit, Eli Lilly and Company, Neuilly-sur-Seine, France; 6 Department of Diabetes, School of Cardiovascular and Metabolic Medicine and Sciences, Faculty of Life Sciences and Medicine, King’s College London, London, UK; 7 Institute of Genetics and Cancer, College of Medicine and Veterinary Medicine, University of Edinburgh, Edinburgh, UK; 8 RW Data Assets, AI & Analytics(AIA), Novo Nordisk A/S, Soeberg, Denmark; 9 Eli Lilly and Company, Indianapolis, IN USA; 10 Systems Medicine, School of Medicine, University of Dundee, Dundee, UK; 11 Division of Endocrinology and Metabolic Disease, Department of Internal Medicine, Maastricht University Medical Center, Maastricht, the Netherlands; 12 Institute of Clinical Medicine, University of Copenhagen, Copenhagen, Denmark; 13 School of Health and Related Research (ScHARR), University of Sheffield, Sheffield, UK

**Keywords:** Consequences, Cox regression, Hypoglycaemia, Hypo-RESOLVE

## Abstract

**Aims/hypothesis:**

Whether hypoglycaemia increases the risk of other adverse outcomes in diabetes remains controversial, especially for hypoglycaemia episodes not requiring assistance from another person. An objective of the Hypoglycaemia REdefining SOLutions for better liVEs (Hypo-RESOLVE) project was to create and use a dataset of pooled clinical trials in people with type 1 or type 2 diabetes to examine the association of exposure to all hypoglycaemia episodes across the range of severity with incident event outcomes: death, CVD, neuropathy, kidney disease, retinal disorders and depression. We also examined the change in continuous outcomes that occurred following a hypoglycaemia episode: change in eGFR, HbA_1c_, blood glucose, blood glucose variability and weight.

**Methods:**

Data from 84 trials with 39,373 participants were pooled. For event outcomes, time-updated Cox regression models adjusted for age, sex, diabetes duration and HbA_1*c*_ were fitted to assess association between: (1) outcome and cumulative exposure to hypoglycaemia episodes; and (2) outcomes where an acute effect might be expected (i.e. death, acute CVD, retinal disorders) and any hypoglycaemia exposure within the last 10 days. Exposures to any hypoglycaemia episode and to episodes of given severity (levels 1, 2 and 3) were examined. Further adjustment was then made for a wider set of potential confounders. The within-person change in continuous outcomes was also summarised (median of 40.4 weeks for type 1 diabetes and 26 weeks for type 2 diabetes). Analyses were conducted separately by type of diabetes.

**Results:**

The maximally adjusted association analysis for type 1 diabetes found that cumulative exposure to hypoglycaemia episodes of any level was associated with higher risks of neuropathy, kidney disease, retinal disorders and depression, with risk ratios ranging from 1.55 (*p*=0.002) to 2.81 (*p*=0.002). Associations of a similar direction were found when level 1 episodes were examined separately but were significant for depression only. For type 2 diabetes cumulative exposure to hypoglycaemia episodes of any level was associated with higher risks of death, acute CVD, kidney disease, retinal disorders and depression, with risk ratios ranging from 2.35 (*p*<0.0001) to 3.00 (*p*<0.0001). These associations remained significant when level 1 episodes were examined separately. There was evidence of an association between hypoglycaemia episodes of any kind in the previous 10 days and death, acute CVD and retinal disorders in both type 1 and type 2 diabetes, with rate ratios ranging from 1.32 (*p*=0.017) to 2.68 (*p*<0.0001). These associations varied in magnitude and significance when examined separately by hypoglycaemia level. Within the range of hypoglycaemia defined by levels 1, 2 and 3, we could not find any evidence of a threshold at which risk of these consequences suddenly became pronounced.

**Conclusions/interpretation:**

These data are consistent with hypoglycaemia being associated with an increased risk of adverse events across several body systems in diabetes. These associations are not confined to severe hypoglycaemia requiring assistance.

**Graphical Abstract:**

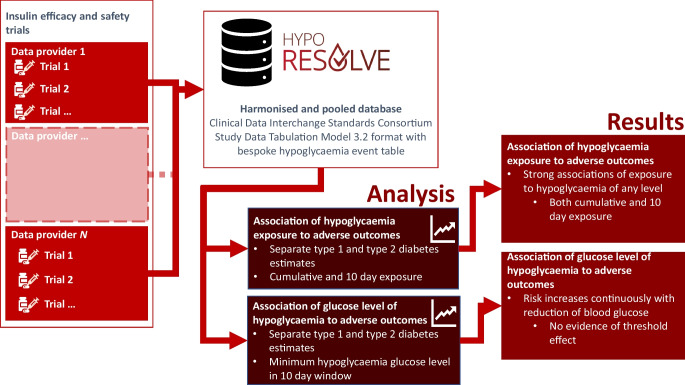

**Supplementary Information:**

The online version contains supplementary material available at 10.1007/s00125-024-06225-1.


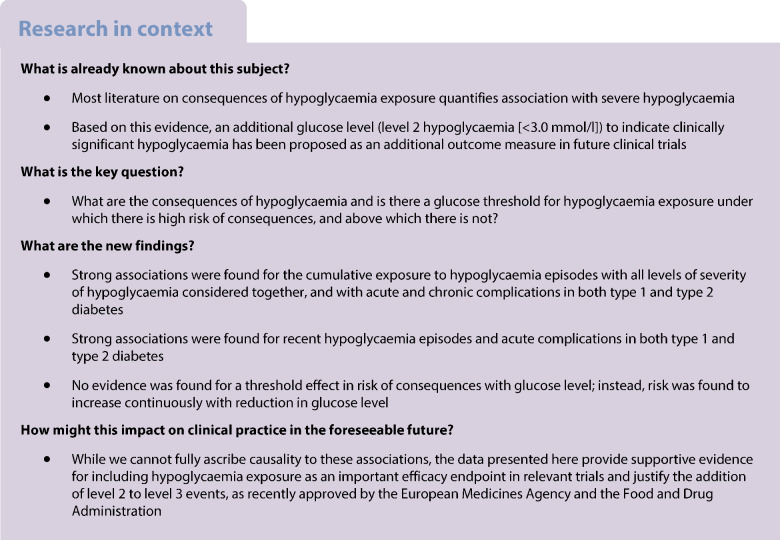


## Introduction

Hypoglycaemia may occur as a consequence of insulin therapy or insulin secretagogue treatment combined with deficiencies in the normal physiological counterregulatory defences [1]. The negative consequences (physical and psychological) of hypoglycaemia present a constant source of concern for people with diabetes and their families [2].

The International Hypoglycaemia Study Group (IHSG) recommended categorising hypoglycaemia episodes into three levels, with level 2 (a glucose level of <3.0 mmol/l [<54 mg/dl]) or level 3 (an episode requiring external assistance for recovery) considered to be sufficiently low to indicate serious, clinically important events that warrant reporting in clinical trials.

More recently, the Food and Drug Administration (FDA) and the European Medicines Agency (EMA) have endorsed level 2 and level 3 events as potential clinical endpoints in clinical trials. These cutoffs for defining levels were largely based on expert opinion, as well as on data from clinical trials and research indicating that glucose levels less than 3.0 mmol/l were associated with the development of acute cognitive impairment, cardiac arrythmias and increased cardiovascular and all-cause mortality, even without hypoglycaemic coma [3–12].

Previous studies in both type 1 and type 2 diabetes have shown associations between, mostly severe, hypoglycaemia exposure and mortality [8, 9, 13–19], CVD [14] (including myocardial infarction), retinal disorder [20], kidney disease [21], neuropathy [21] and depression [22]. Many of these studies examined associations only with level 2 or 3 hypoglycaemia, and were small in scale with substantial scope for residual confounding by factors such as higher mean glucose control and hypertension [23]. Large, pooled datasets on the association of robustly assessed hypoglycaemia with adverse outcomes, albeit from a randomised controlled trial setting, should provide supporting evidence for the use of hypoglycaemia as an acceptable efficacy endpoint in relevant trials and also enable economic analyses of the potential wider benefits of hypoglycaemia prevention.

The EU-funded Hypoglycaemia REdefining SOLutions for better liVEs (Hypo-RESOLVE) project brought people with diabetes together with academic, clinical and industry partners with the goal of identifying and quantifying predictors and consequences of hypoglycaemia [1]. As part of this initiative, a single database was created based on insulin clinical trial data provided by several pharmaceutical industry partners from people with type 1 or type 2 diabetes in whom hypoglycaemia events occurred for the duration of the trials. We leveraged this dataset to examine the prospective association of exposure to hypoglycaemia episodes of any level and varying severity with a range of clinical outcomes captured during the trials.

## Methods

### Data and cohort

Data from 25 clinical trials involving 11,392 people living with type 1 diabetes and 59 trials involving 27,981 people living with type 2 diabetes were provided by industry partners. All trials involved people with diabetes who were taking glucose-lowering medication, mostly insulin either alone or in combination with oral drugs, with hypoglycaemia risk. The raw trial data were standardised, harmonised and pooled in a database using the Clinical Data Interchange Consortium (CDISC) Study Data Tabulation Model (SDTM 3.2) format [24] (see the electronic supplementary material [ESM] Methods for details). In addition, the bespoke domain XH was created for hypoglycaemia event data, obtained from participant diaries and Medical Dictionary for Regulatory Activities (MedDRA) serious adverse event declaration from clinical trials. The trials did not use continuous glucose monitoring. Some episodes will have been asymptomatic episodes noted on self-monitored blood glucose that met the agreed thresholds for hypoglycaemia, and some will have been symptomatic episodes. Level 3 episodes did not require a blood glucose as this is not part of the definition, but one would often be recorded. Some level 3 episodes will have been derived also from serious adverse event reporting. Self-monitored blood glucose cycles varied between trials, with a median recorded profile being a four-point profile, although often participants would be asked to do seven-point profiles at specified periods, such as a week leading to a visit. Each hypoglycaemic event was characterised by an event date, a blood glucose measurement (if available) and self-treatment status.

Despite the availability of raw data from each clinical trial, many trials had idiosyncratic data structures or collection procedures which precluded data harmonisation into the pooled database. Some trials will have excluded people with unawareness of hypoglycaemia.

We excluded individuals who: did not pass trial screening; lacked observation start or end dates; had missing age, sex (which was reported by the investigator of the clinical trial) or diabetes duration information; or had more than 20% missingness for hypoglycaemic event data. A hypoglycaemic event was considered missing if the event lacked a date of occurrence, or it lacked a glucose measurement while simultaneously either being denoted as a self-treated event or the self-treatment status was missing.

### Definitions of hypoglycaemia

The pooled clinical trial dataset contained the blood glucose measurement and whether assistance was required to treat the episode for each hypoglycaemic event. This information was used to define hypoglycaemia in our analyses, irrespective of how hypoglycaemia had been defined in each contributing trial in the pooled dataset.

The IHSG [25] proposed three levels of hypoglycaemia which have been largely accepted recently by the EMA [26] and, as draft guidance, by the FDA [27]. These are:

- level 1 hypoglycaemia alert events, defined as any event with a recorded blood glucose level of less than 3.9 mmol/l but not less than 3.0 mmol/l
- level 2 hypoglycaemic events, defined as any hypoglycaemic event with a recorded blood glucose level below 3.0 mmol/l
- level 3 hypoglycaemic events (severe hypoglycaemia [SH]), defined as any hypoglycaemic event in which the individual was unable to self-treat due to severe cognitive impairment, irrespective of glucose measurement

Within the pooled clinical trial dataset, level 3 was any event in the XH table that was both symptomatic and not self-treated.

### Definitions of hypoglycaemia exposure

We tested whether there was evidence of an association between outcome and the following exposures:

1. exposure to a recent hypoglycaemia episode i.e. within the last 10 days (an arbitrary period within which we could reasonably expect an acute response to hypoglycaemia to manifest)
2. cumulative exposure to hypoglycaemia episodes

Associations of exposure to any hypoglycaemia episode and to episodes of given severity (levels 1, 2 and 3) separately were examined.

### Outcomes

Our aim was to evaluate the prospective association of hypoglycaemia exposure with a range of outcomes. Outcomes for consideration were informed by an expert panel (including clinicians, people with diabetes and other stakeholders), a systematic literature review and data availability. Event outcomes were death, incident acute CVD, incident retinal disorders, incident neuropathy, incident kidney disease and incident depression. Adverse event outcomes were ascertained by the adverse event reporting system in the trials and defined by relevant MedDRA preferred terms (see ESM Table 1). These could be either worsening or new occurrences reported as adverse or serious adverse events depending on severity. Such events rely on reporting both at routine follow-up visits and/or because of acute presentation of the trial participant. Quantitative outcomes were captured at routine follow-up visits and included HbA_1c_, blood glucose, eGFR as defined by the Chronic Kidney Disease Epidemiology Collaboration (CKD-EPI) equation [28] and body weight. We also derived a commonly used measure of blood glucose variability, i.e. the CV calculated as the ratio of the standard deviation to the mean of blood glucose within a 6 week time interval, with the interval duration chosen arbitrarily in which to estimate a typical CV baseline.

### Missingness, evaluability and imputation

All continuous covariates could be categorised either as having an evaluable continuous value or as being missing. For categorical covariates such as sex and ethnicity (collected by their physician), the covariate was considered either evaluable or missing. For drug exposure and medical history covariates, if at least one person in a given trial had the covariate recorded, we considered all the participants in that trial to be evaluable for these covariates, and otherwise we regarded the covariates as non-evaluated in a given trial.

Covariates were imputed on a per-trial basis using the R package Amelia (version 1.7.6, https://cran.r-project.org/web/packages/Amelia/index.html), provided the covariate was present for at least 80% of participants in that trial.

### Statistical methods

Analyses were performed separately for type 1 and type 2 diabetes.

#### Adverse event outcome model construction

We fitted Cox proportional-hazards models to time-to-incident-event outcomes using the coxph function of the R package survival. Individuals entered the study at randomisation and exited the study at the earliest of date of death, end of participation in the clinical trial or event outcome of interest. Exposures were time-updated with the time scale of the Cox model given as days since study start. Adjustment covariates were given as their value at the earliest available date. Minimally adjusted models were adjusted for study identifier, age, sex, diabetes duration and HbA_1*c*_. Fully adjusted models were adjusted additionally for a set of covariates selected from risk factors that we found were associated with future hypoglycaemia across types of diabetes: insulin regimen (premix, basal-bolus or basal, and excluding use of continuous subcutaneous insulin infusion where the number of individuals was small), insulin origin (analogue, human or human+analogue), daily insulin dose, diastolic BP, ethnicity, self-monitored blood glucose, self-monitored blood glucose CV, medical history of complications (CVD/retinopathy/neuropathy/nephropathy status) and antihypertensive/anti-inflammatories/psychoactives/antiepilepsy drug/blood glucose-lowering medication use. Medical history covariates were defined by relevant MedDRA terms, and drug categories were defined using Anatomical Therapeutic Chemical (ATC) codes (see ESM Medical history definitions and Concomitant medications definitions in ESM Tables 2 and 3, respectively). For each outcome, each covariate was included in the model if its missingness did not reduce the number of incident events used in analysis by more than 30%. See ESM Tables 4 and 5 for the final covariates included for each analysis. Trials in which the outcome of interest was not observed were not included in analysis as they contributed no information on rate ratios. Incidence rate ratios of fitted models were assessed. Two time scales of exposure were considered: across the full trial period (median trial duration of 40.4 weeks for type 1 diabetes and 26 weeks for type 2 diabetes), and the immediate effect captured by the relative risk associated with recent exposure to hypoglycaemia in the preceding 10 days. We chose an arbitrary time window of 10 days to show the likely maximal immediate effect. Such analyses of immediate effect were restricted to events reasonably hypothesised to be acutely affected by hypoglycaemia (death, acute CVD and retinal disorders, but not neuropathy or nephropathy or depression). For longer-term analysis we encoded hypoglycaemia exposure in our models as three separate cumulative counts, one cumulative count for each of level 1, level 2 and level 3 hypoglycaemia observed. Cumulative exposures were transformed by incrementing by one and then applying the natural log to account for skew in the exposure distributions. For recent analysis total exposure within a 10 day window was used, with levels 1, 2 and 3 coded as separate covariates. For both time scales we also fit models that coded hypoglycaemia of any level as a single covariate, as opposed to at levels 1, 2 and 3 separately.

In order to examine the form of the relationship between blood glucose values and outcomes, and in particular to evaluate whether there is evidence for any specific glucose threshold within the accepted hypoglycaemia range at which risk of outcome suddenly rises, we also fitted Cox models where the exposure was defined and time-updated as the minimum blood glucose occurring in any hypoglycaemic episode during a 10 day window. In cases when there was no hypoglycaemic episode within the 10 day window the minimum blood glucose exposure was set to 4.0 mmol/l and for level 3 hypoglycaemic events where the blood glucose was not available then the minimum blood glucose exposure was assumed to be very low and set to 2.4 mmol/l. The exposure was included using the R function pspline with 2 degrees of freedom.

## Results

### Data availability

Data, after exclusion criteria were applied, consisted of 11,392 people from 25 clinical trials with type 1 diabetes and 27,981 people from 59 trials with type 2 diabetes. During follow-up there were a total of 841,397 and 299,623 level 1 or worse hypoglycaemic events in the type 1 and type 2 diabetes cohorts, respectively. For level 2 or worse there were 334,085 and 72,600 hypoglycaemic events in the type 1 and type 2 diabetes cohorts, respectively, and finally for level 3 there were 4719 and 3390 events in the type 1 and type 2 diabetes cohorts, respectively.

### Cohort characteristics

Characteristics of the cohort by type of diabetes are presented in Tables 1 and 2. The median age was 38 years in type 1 diabetes and 59 years in type 2 diabetes participants. Almost half of all participants were female. The vast majority were white. Very few of the type 1 diabetes participants had CVD at baseline but 18.8% of those with type 2 diabetes did. Those with type 1 diabetes had longer diabetes duration and higher frequency of reported microvascular complications at baseline. Tables 1 and 2 also show the numbers of evaluable people after imputation for each covariate.

**Table 1:** Cohort characteristics for individuals with type 1 diabetes in the first 6 weeks from trial entry

Covariate	Median (IQR)/*n* (%)	Evaluable participants	Evaluable studies
Age (yr)	38 (26–50)	11,392	25
Female	5017 (44.04)	11,392	25
Ethnicity: non-white	1505 (14.25)	10,564	22
Diabetes duration (yr)	13.50 (6.10–23.42)	11,392	25
HbA_1c_ (mmol/mol)	61.75 (54.65–69.41)	11,236	24
HbA_1c_ (%)	7.8 (7.15–8.50)	11,236	24
Blood glucose (mmol/l)	8.76 (7.65–0.04)	10,217	22
eGFR (ml/min per 1.73 m^2^)	103.68 (88.49–120.43)	10,106	21
Systolic BP (mmHg)	120.67 (111.00–130.67)	11,392	25
HDL-cholesterol (mmol/l)	1.58 (1.31–1.92)	8426	16
Triglycerides (mmol/l)	0.88 (0.66–1.23)	7242	14
Total daily insulin dose (U/day)	44 (27.81–64.25)	11,129	24
Insulin origin: human	249 (2.25)	11,044	24
Insulin origin: analogue	10,026 (90.78)	11,044	24
Insulin origin: human+analogue	769 (6.96)	11,044	24
Insulin regimen: premix	0 (0)	11,044	24
Insulin regimen: basal-bolus	11,044 (100)	11,044	24
Insulin regimen: basal	0 (0)	11,044	24
CVD at baseline	401 (3.52)	11,392	25
Retinopathy at baseline	2960 (26.81)	11,041	24
Neuropathy at baseline	431 (4.29)	10,047	22
Nephropathy at baseline	1814 (18.99)	9551	20
CM: psychoactive drugs	1127 (10.13)	11,129	24
CM: BG-lowering	36 (0.43)	8445	15
CM: anti-epileptic	214 (2.01)	10,640	22
CM: anti-thyroid drugs	49 (0.51)	9597	18

Median and IQR are reported for continuous variables. IQR is given as the distance between the 25th and 75th percentiles

Reported values are calculated across time-updated 6 week person–time intervals

See the ESM for CVD MedDRA preferred terms

Frequency and percentage of those evaluable are reported for categorical variables

BG, blood glucose; CM, concomitant medication

**Table 2:** Cohort characteristics for individuals with type 2 diabetes during the first 6 weeks from trial entry

Covariate	Median (IQR)/*n* (%)	Evaluable participants	Evaluable studies
Age (yr)	59.00 (52.15–66.00)	27,981	59
Female	13,019 (46.53)	27,981	59
Ethnicity: non-white	8120 (32.33)	25,118	53
Diabetes duration (yr)	11 (6.70–16.10)	27,981	59
HbA_1c_ (mmol/mol)	65.03 (58.47–73.78)	27,570	57
HbA_1c_ (%)	8.1 (7.5–8.9)	27,570	57
Blood glucose (mmol/l)	8.04 (6.85–9.46)	26,535	53
eGFR (ml/min per 1.73 m^2^)	88.37 (73.12–99.21)	26,179	53
Systolic BP (mmHg)	131.67 (122.67–141.00)	27,133	57
HDL-cholesterol (mmol/l)	1.17 (0.98–1.40)	20,664	42
Triglycerides (mmol/l)	1.56 (1.12–2.21)	20,664	42
Total daily insulin dose (U/day)	31 (20.20–60.32)	27,406	56
Insulin origin: human	1381 (5.11)	27,033	57
Insulin origin: analogue	24,372 (90.16)	27,033	57
Insulin origin: human+analogue	420 (1.55)	27,033	57
Insulin regimen: premix	3403 (12.84)	26,499	56
Insulin regimen: basal-bolus	7969 (30.07)	26,499	56
Insulin regimen: basal	15,127 (57.09)	26,499	56
CVD at baseline	4831 (18.8)	25,691	54
Retinopathy at baseline	4778 (19.51)	24,490	52
Neuropathy at baseline	1608 (6.8)	23,639	49
Nephropathy at baseline	3665 (16.05)	22,833	46
CM: psychoactive drugs	4051 (15.43)	26,259	55
CM: BG-lowering	20,708 (82.52)	25,096	52
CM: anti-epileptic	944 (3.66)	25,815	53
CM: anti-thyroid drugs	69 (0.34)	20,279	37

Reported values are calculated across time-updated 6 week person–time intervals

Median and IQR are reported for continuous variables. IQR is given as the distance between the 25th and 75th percentiles

See the ESM for CVD MedDRA preferred terms

Frequency and percentage of those evaluable are reported for categorical variables

BG, blood glucose; CM, concomitant medication

### Association of hypoglycaemia episodes with adverse event outcomes

#### Association with cumulative exposure

As shown in Table 3, for participants with type 1 diabetes, we found that greater cumulative exposure to hypoglycaemia episodes of any level was associated with higher risks of acute CVD, neuropathy, kidney disease (predominantly but not exclusively diabetic), retinal disorders and depression, with risk ratios in fully adjusted models ranging from 1.55 (*p*=0.002) to 2.81 (*p*=0.002). There were 21 deaths overall in those with type 1 diabetes with no significant association with hypoglycaemia events detected. The full adjustment for covariates made little difference to the magnitude of the associations, although the association with acute CVD became non-significant. We fit models adjusted for time-updated HbA_1c_ and found little difference to those fit using baseline measures. For example, for cumulative all-level hypoglycaemia exposure the risk ratio with acute CVD in type 1 diabetes changed from 1.593 (95% CI 1.096, 2.317) to 1.57 (95% CI 1.078, 2.285).

**Table 3:** Rate ratios giving the increase in rate of outcome event for every standard deviation increase in log(1 + cumulative exposure to all hypoglycaemia) for various outcomes for type 1 diabetes

Adverse event	Individuals	Events	Hypoglycaemia of any level	
			Total exposure	Rate ratio
Minimally adjusted				
Death	4901	21	474,031	1.116 (0.716, 1.740)
CVD	9486	90	787,600	1.593 (1.096, 2.317)^a^
Neuropathy	7362	42	593,915	3.048 (1.837, 5.057)^a^
Kidney disease	8265	60	729,037	1.369 (1.063, 1.762)^a^
Retinal disorders	9744	328	797,095	1.977 (1.706, 2.291)^a^
Depression	10,232	135	825,202	2.568 (1.808, 3.647)^a^
Fully adjusted				
Death	4765	21	466,710	1.023 (0.698, 1.499)
CVD	6823	73	671,674	1.479 (0.961, 2.278)
Neuropathy	5292	35	497,730	2.814 (1.482, 5.342)^a^
Kidney disease	6083	54	630,035	1.546 (1.180, 2.026)^a^
Retinal disorders	6665	240	657,094	1.874 (1.556, 2.256)^a^
Depression	7750	110	720,892	2.651 (1.806, 3.892)^a^

Rate ratios are given with 95% CIs

Total exposure is the total hypoglycaemic exposure for the given hypoglycaemia level

Definitions of adverse events in terms of MedDRA preferred terms can be found in the ESM

Adjustment covariates for fully adjusted models can be found in the ESM

^a^Statistically significant rate ratios

For the acute CVD outcome, we added interaction terms to the minimally adjusted model between study identifier and cumulative exposure to hypoglycaemia and tested for heterogeneity using a χ^2^ test for the difference in deviance between the models with and without the interaction terms. From the test, we found there was heterogeneity (*p*<0.00001) where the IQR of risk ratios for increased cumulative exposure on acute CVD was 1.04–2.11.

As shown in Table 4, the CIs for the rate ratios for events were much wider when tested in a model with cumulative counts of level 1, 2 and 3 episodes entered as separate terms. With the exception of acute CVD, although there was a similar direction of association of the outcomes with level 1 and 2 episodes, the fully adjusted model found statistically significant associations at *p*=10^−4^ only for depression for level 1 and retinal disorders for level 2. The power to detect associations with the most infrequent level 3 events independently of effects of levels 1 and 2 was limited. Estimates for adverse outcomes with sparse events may be unstable, especially with full adjustment of models.

**Table 4:** Rate ratios giving the increase in rate of outcome event for every standard deviation increase in log(1 + cumulative exposure to hypoglycaemia) for various outcomes for type 1 diabetes

Adverse event	Individuals	Events	Level 1		Level 2		Level 3	
			Total exposure	Rate ratio	Total exposure	Rate ratio	Total exposure	Rate ratio
Minimally adjusted								
Death	4901	21	292,452	0.666 (0.264, 1.676)	179,172	1.824 (0.708, 4.702)	2411	0.686 (0.311, 1.513)
CVD	9486	90	474,152	0.922 (0.451, 1.887)	309,928	1.656 (0.911, 3.011)	3526	1.188 (0.918, 1.536)
Neuropathy	7362	42	364,484	1.556 (0.750, 3.230)	227,085	1.952 (0.977, 3.902)	2350	1.094 (0.848, 1.411)
Kidney disease	8265	60	447,757	1.266 (0.731, 2.191)	276,737	1.054 (0.618, 1.798)	4613	0.902 (0.527, 1.544)
Retinal disorders	9744	328	477,840	1.489 (1.161, 1.911)^a^	314,480	1.497 (1.243, 1.803)^a^	4846	0.941 (0.783, 1.131)
Depression	10,232	135	506,369	2.746 (1.397, 5.399)^a^	315,268	1.269 (0.845, 1.906)	3571	0.808 (0.594, 1.099)
Fully adjusted								
Death	4765	21	288,356	0.668 (0.274, 1.628)	175,957	1.736 (0.703, 4.282)	2401	0.626 (0.263, 1.491)
CVD	6823	73	413,050	1.106 (0.535, 2.286)	255,228	1.361 (0.766, 2.42)	3401	1.131 (0.856, 1.494)
Neuropathy	5292	35	305,399	1.719 (0.910, 3.247)	190,056	1.735 (0.865, 3.479)	2278	1.098 (0.866, 1.393)
Kidney disease	6083	54	394,305	1.503 (0.792, 2.853)	232,539	1.056 (0.597, 1.869)	3195	0.817 (0.452, 1.475)
Retinal disorders	6665	240	404,748	1.241 (0.913, 1.687)	249,014	1.562 (1.235, 1.975)^a^	3336	0.946 (0.785, 1.140)
Depression	7750	110	445,668	2.762 (1.651, 4.622)^a^	271,748	1.27 (0.82, 1.967)	3481	0.755 (0.523, 1.09)

Rate ratios are given with 95% CIs

Definitions of adverse events in terms of MedDRA preferred terms can be found in the ESM

Adjustment covariates for fully adjusted models can be found in the ESM

^a^Statistically significant rate ratios

For type 2 diabetes, as shown in Table 5, cumulative exposure to hypoglycaemia episodes of any level was associated with higher risks of death, acute CVD, kidney disease, retinal disorders and depression, with risk ratios in fully adjusted models ranging from 2.35 (*p*<0.0001) to 3.00 (*p*<0.0001). The full adjustment for covariates made little difference to the associations. As shown in Table 6, the CIs for the rate ratios for events were much wider when tested in a model with the cumulative counts of level 1, 2 and 3 episodes entered as separate terms, but significant independent associations for all outcomes were seen in fully adjusted models for level 1 and for acute CVD, kidney disease and depression for level 2. CIs for level 3 were wide and no significant associations were detectable. For type 2 diabetes, we further included an interaction term between CVD at baseline and cumulative hypoglycaemia of any level and found no association with acute CVD (*p*>0.2 for both minimally and fully adjusted models).

**Table 5:** Rate ratios giving the increase in rate of outcome event for every standard deviation increase in log(1 + cumulative exposure to all hypoglycaemia) for various outcomes for type 2 diabetes

Adverse event	Individuals	Events	Hypoglycaemia of any level	
			Total exposure	Rate ratio
Minimally adjusted				
Death	15,408	92	202,600	2.497 (1.862, 3.350)^a^
CVD	27,009	719	296,126	2.942 (2.465, 3.511)^a^
Neuropathy	21,498	138	258,492	2.539 (1.746, 3.691)^a^
Kidney disease	23,150	203	275,034	2.604 (2.001, 3.387)^a^
Retinal disorders	26,706	679	292,838	2.713 (2.341, 3.144)^a^
Depression	23,875	215	259,997	2.52 (1.912, 3.321)^a^
Fully adjusted				
Death	14,195	89	199,757	2.345 (1.696, 3.243)^a^
CVD	20,662	584	272,442	2.996 (2.496, 3.596)^a^
Neuropathy	17,125	112	244,019	2.383 (1.626, 3.493)^a^
Kidney disease	18,507	160	259,929	2.662 (2.009, 3.526)^a^
Retinal disorders	20,481	585	271,880	2.635 (2.269, 3.060)^a^
Depression	17,843	167	237,733	2.348 (1.756, 3.139)^a^

Rate ratios are given with 95% CIs

Total exposure is the total hypoglycaemic exposure for the given hypoglycaemia level

Definitions of adverse events in terms of MedDRA preferred terms can be found in the ESM

Adjustment covariates for fully adjusted models can be found in the ESM

^a^Statistically significant rate ratios

**Table 6:** Rate ratios giving the increase in rate of outcome event for every standard deviation increase in log(1 + cumulative exposure to hypoglycaemia) for various outcomes for type 2 diabetes

Adverse event	Individuals	Events	Level 1		Level 2		Level 3	
			Total exposure	Rate ratio	Total exposure	Rate ratio	Total exposure	Rate ratio
Minimally adjusted								
Death	15,408	92	154,162	1.703 (0.983, 2.950)	47,444	1.44 (0.732, 2.835)	1014	0.875 (0.568, 1.348)
CVD	27,009	719	227,472	2.097 (1.754, 2.508)^a^	67,064	1.426 (1.145, 1.777)^a^	1630	1.116 (0.959, 1.299)
Neuropathy	21,498	138	198,163	1.759 (1.138, 2.720)^a^	58,831	1.475 (0.994, 2.189)	1533	1.104 (0.735, 1.656)
Kidney disease	23,150	203	212,914	1.768 (1.197, 2.610)^a^	60,603	1.490 (0.998, 2.225)	1556	1.003 (0.820, 1.227)
Retinal disorders	26,706	679	224,997	2.677 (2.268, 3.161)^a^	66,262	1.061 (0.896, 1.257)	1615	0.925 (0.765, 1.117)
Depression	23,875	215	200,429	1.762 (1.313, 2.366)^a^	58,056	1.509 (1.132, 2.012)^a^	1551	1.026 (0.862, 1.220)
Fully adjusted								
Death	14,195	89	151,967	1.709 (1.016, 2.876)^a^	46,819	1.320 (0.701, 2.482)	990	0.873 (0.567, 1.343)
CVD	20,662	584	209,233	2.252 (1.859, 2.729)^a^	61,711	1.339 (1.108, 1.618)^a^	1537	1.121 (0.977, 1.287)
Neuropathy	17,125	112	186,923	1.798 (1.129, 2.864)^a^	55,634	1.333 (0.901, 1.972)	1496	1.035 (0.675, 1.588)
Kidney disease	18,507	160	200,338	1.735 (1.165, 2.583)^a^	58,159	1.535 (1.030, 2.288)^a^	1470	0.995 (0.812, 1.221)
Retinal disorders	20,481	585	208,758	2.728 (2.290, 3.251)^a^	61,593	0.997 (0.834, 1.193)	1564	0.866 (0.693, 1.082)
Depression	17,843	167	182,901	1.839 (1.361, 2.484)^a^	53,401	1.335 (1.015, 1.755)^a^	1469	0.961 (0.797, 1.159)

Rate ratios are given with 95% CIs

Total exposure is the total hypoglycaemic exposure for the given hypoglycaemia level

Definitions of adverse events in terms of MedDRA preferred terms can be found in the ESM

Adjustment covariates for fully adjusted models can be found in the ESM

^a^Statistically significant rate ratios

For the acute CVD outcome we tested for heterogeneity, similarly as stated above for type 1 diabetes, and found there was heterogeneity (*p*<0.00001) where the IQR of risk ratios for increased cumulative exposure on acute CVD was 1.69–3.43.

Associations of event with exposure to any hypoglycaemia episodes in the previous 10 days were found for death, acute CVD and retinal disorders in both types of diabetes with rate ratios ranging from 1.32 (*p*=0.017) to 2.68 (*p*<0.0001). In type 1 diabetes, associations were found between acute CVD and levels 2 and 3 and between retinal disorders and levels 1 and 2. In type 2 diabetes, associations were found between both death and retinal disorders and levels 1 and 2 and between acute CVD and all levels (ESM Tables 6–9 and ESM Association of events with exposure with hypoglycaemia episodes in the previous 10 days). For type 2 diabetes, we further included an interaction term between CVD at baseline and hypoglycaemia of any level in the previous 10 days and found evidence that more hypoglycaemia concurrently with CVD at baseline led to increased risk in acute CVD for the minimally adjusted model (*p*=0.027) and the fully adjusted model (*p*=0.040).

We found no evidence of pre- to post-hypoglycaemic episode hypoglycaemia changes in continuous measures (see ESM Analysis of change in continuous outcomes and ESM Figs 1–3).

#### Assessment of the form of the association of blood glucose during hypoglycaemic episodes with events

**Fig. 1:**
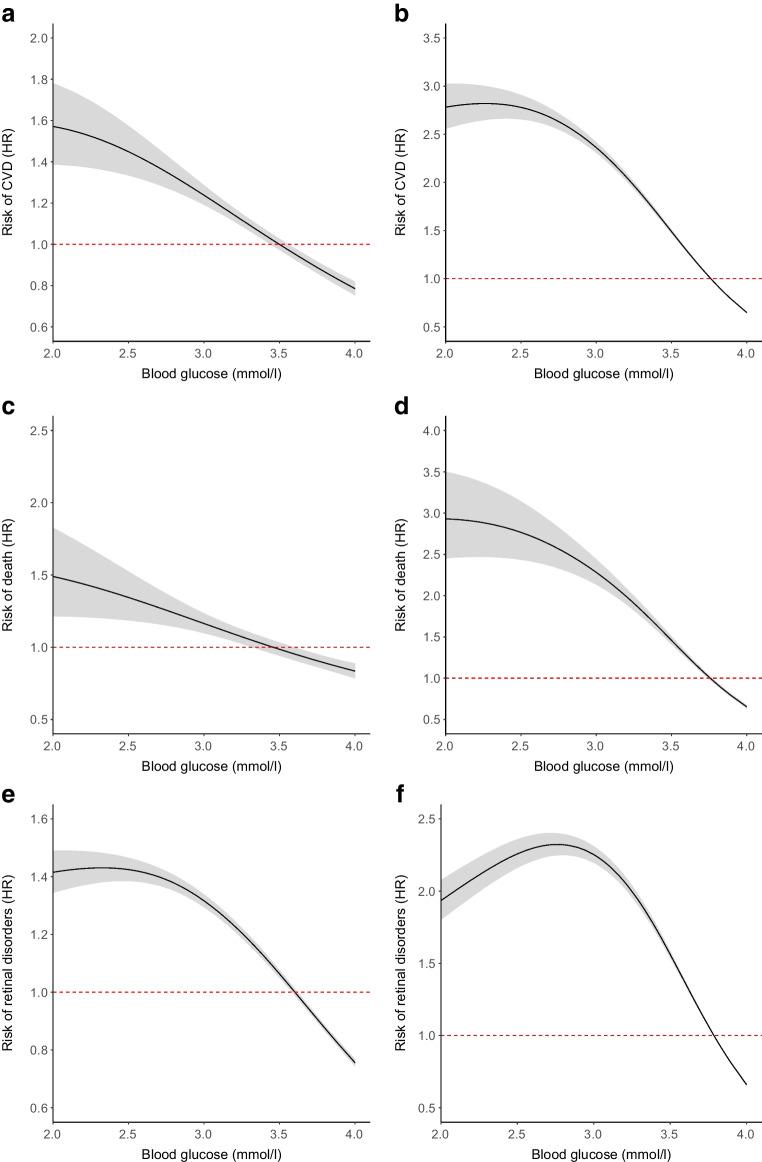
How rate ratios of outcomes change with respect to hypoglycaemia exposure as determined by minimum glucose level of a hypoglycaemic episode within a 10 day period. Panels correspond to outcomes as follows: (**a**) type 1 diabetes CVD, (**b**) type 2 diabetes CVD, (**c**) type 1 diabetes death, (**d**) type 2 diabetes death, (**e**) type 1 diabetes retinal disorders and (**f**) type 2 diabetes retinal disorders

Figure 1 shows the modelled relationship of how the risk of death, acute CVD and retinopathy varies with the minimum glucose level associated in the preceding 10 day period, with the blood glucose value set at 4 mmol/l in the absence of a hypoglycaemic episode and set at 2.4 mmol/l for level 3 episodes with no blood glucose recorded. This model suggests that for both types of diabetes the association with each of these outcomes is a continuous one with no evidence of a threshold point where risk abruptly increases. As shown by the different scales of the *y* axes, risk ratios were higher for type 2.

In both type 1 and type 2 diabetes, analysis of association between acute CVD and preceding 10 day minimum glucose level was repeated with an additional interaction term between HbA_1c_ and the minimum glucose level. No interaction effect was observed (*p*=0.78 for type 1 and *p*=0.84 for type 2).

## Discussion

The Hypo-RESOLVE database brought together prospective data on incident events following hypoglycaemia in type 1 and type 2 diabetes from clinical trials of glucose-lowering agents (all trials included insulin). We found strong associations for the cumulative exposure to total hypoglycaemia episodes at any level of severity with death, incident CVD, retinopathy and chronic complications in type 2 diabetes, and with incident CVD, retinopathy and chronic complications in type 1 diabetes. There was no interaction effect between CVD at baseline and the effect of cumulative hypoglycaemia on acute CVD. There were strong associations of recent hypoglycaemia episodes with death, acute CVD and retinopathy events. We also found recent hypoglycaemia exposure in those with CVD at baseline was associated with more acute CVD. This may reflect a greater vulnerability of an already damaged myocardium to the effects of hypoglycaemia. For type 1 diabetes, fully adjusted associations with death, of which there were few in this younger population, and acute CVD were significant only for recent exposure to hypoglycaemia. These data reinforce evidence from the literature that more frequent hypoglycaemia is at least a risk marker for future adverse outcomes in people with diabetes. Regarding causality, we adjusted for possible confounders of these associations and this adjustment had little impact on the magnitude of associations seen, which is consistent with, but not proof of, these associations being causal.

An important clinical consideration is whether different biochemical levels of hypoglycaemia have different associations with outcomes. Due to correlation between numbers of level 1, 2 and 3 events, it is difficult to discern independent associations of level 2 and level 3 episodes beyond level 1 episodes (level 1 to level 2, Spearman ρ 0.67; and level 2 to level 3, Spearman ρ 0.22). This does not mean that level 3 hypoglycaemia is not associated with these outcomes. Within the hypoglycaemia range the data were consistent with a continuous relationship of increasing risks of events with lower blood glucose rather than with a blood glucose threshold effect below which risk suddenly rose. This analysis only considered the truncated distribution of blood glucose up to 4 mmol/l for which we have hypoglycaemia events and therefore does not imply higher glucose values are unimportant for complications. We found no evidence of pre- to post-hypoglycaemic episode hypoglycaemia changes in continuous measures (see ESM Analysis of change in continuous outcomes).

### Comparison with existing literature

Research across type 1 and type 2 diabetes cohorts has consistently shown an increased mortality risk following hypoglycaemia. For type 2 diabetes, there is a well-documented association between level 3 hypoglycaemia and increased all-cause mortality risk [3–10]. While fewer studies address levels 1 and 2, the LEADER study [19] identified a link between 12 or more annual non-severe hypoglycaemic episodes (<3.1 mmol/l or <56 mg/dl) and increased cardiovascular and overall mortality. Similarly, Spanakis et al [11] found SH and hypoglycaemia <3.9 mmol/l (<70 mg/dl) were associated with a higher mortality risk. Lee et al [12] also reported increased cardiovascular and all-cause mortality with frequent hypoglycaemia <3.9 mg/mmol. This mortality risk association with hypoglycaemia varies over time [4, 29]. In type 1 diabetes, studies reveal an increased all-cause mortality risk following level 3 hypoglycaemia [4, 14, 30].

There is also prior evidence for an elevated risk of a range of cardiovascular events with increased hypoglycaemia exposure. In individuals with type 2 diabetes, most studies report level 3 hypoglycaemia outcomes, with some studies showing an association with increased risk of a heart failure [8, 9, 31], but some showing no association [3, 6]. Yun et al [9] showed an association between level 3 hypoglycaemia and increased risk of myocardial infarction, while others showed no association [8, 10]. Wang et al [32] showed an increased risk of coronary heart disease with hypoglycaemia ≤3.9 mmol/l, and the LEADER study [19] showed increased risk of major cardiovascular events with ≥12 non-severe hypoglycaemic events per year. Studies in individuals with type 1 diabetes show increased risk of non-obstructive coronary artery disease and no association for obstructive coronary artery disease [33] or coronary disease [21], with evidence to suggest risk varies over time [4].

The link between cumulative hypoglycaemia exposure and diabetes-related microvascular complications (neuropathy, retinopathy, nephropathy), typically associated with chronic hyperglycaemia exposure in both type 1 and 2 diabetes, was a surprising discovery that could involve both direct and indirect mechanisms. Data are derived from numerous randomised controlled trials, often relatively short in duration, where significant improvements in glycaemic control from baseline are common. Short-term improvement in glycaemic control may temporarily exacerbate underlying microvascular disease [34] and correlate with more frequent hypoglycaemia, suggesting an indirect association. Alternatively, recurrent hypoglycaemia might exacerbate cellular damage incurred by chronic hyperglycaemia exposure [35]. Hyperglycaemia results in oxidative stress and inflammation [36] and impairs cellular antioxidant responses [37–39]. Acute hypoglycaemia acts as a proinflammatory stimulus and induces oxidative stress [40]. Research demonstrates that neuronal superoxide production and oxidative damage increase with rising glucose levels during recovery from hypoglycaemia [41]. Additionally, diabetes and recurrent hypoglycaemia synergistically impair mitochondrial function and promote oxidative damage in rodents [42–44]. This might indicate that diabetes diminishes cellular resilience against hypoglycaemia-induced oxidative stress. Cumulative hypoglycaemia exposure exacerbates the impact of chronic hyperglycaemia on diabetic microvascular complications.

Associations between exposure to hypoglycaemia in the previous 10 days and an increased risk of mortality, acute CVD and retinal disorders in both type 1 and type 2 diabetes were notable. Acute hypoglycaemia initiates numerous proinflammatory responses [45–47], including increases in immune cell numbers [48–50], a phenotypic shift towards more proinflammatory non-classical monocytes, and enhanced cytokine production and release of proinflammatory proteins. These responses are observed in individuals regardless of their diabetes status, level of prior glucose control or awareness of hypoglycaemia [48–50], and can persist up to 7 days post-hypoglycaemia [47, 48]. Additionally, hypoglycaemia induces an acute and persistent prothrombotic effect for at least 7 days [45]. These findings indicate that hypoglycaemia may increase short- and medium-term cardiovascular mortality risk by promoting a sustained systemic proinflammatory and prothrombotic state.

Hypo-RESOLVE sought evidence for the IHSG-driven definitions of hypoglycaemia. Our data have shown the impact, and evidence for differences between levels, of hypoglycaemia. In type 1 diabetes, our fully adjusted models show significance only for level 1 with depression and level 2 with retinopathy. It is plausible that the frequency of, often symptomatic, level 1 events contributes to depression while biological damage occurs at lower glucose levels. This is in keeping with IHSG definitions and supports regulatory bodies’ decisions to include level 2 and 3 hypoglycaemia in clinically important outcomes for clinical trials. Higher risk ratios at all levels in type 2, and the importance of patient-reported outcomes for both types of diabetes, suggest including all three levels as potential outcomes in clinical trials. The IHSG in their position statement argued that level 1 hypoglycaemia might be included depending on the trial purpose. Evidence that level 1 hypoglycaemia is associated with depression suggests that its inclusion as an endpoint would be particularly relevant to studying relationships between hypoglycaemia, mental health and quality of life.

### Strengths and limitations of this analysis

This analysis used several different methods and the dataset was large for type 2 diabetes, although the number of events observed for type 1 diabetes was small, as expected, due to the younger age of this participant group. The trial durations were relatively short to determine consequences of hypoglycaemia, where large long-term CVD studies would be preferable but were not available.

There was a high level of missingness for some variables requiring imputation, and some important potential confounders of the association between hypoglycaemia episodes and events were not available. Thus, there is strong potential for residual confounding in these associations as well as potential for ascertainment bias. We have followed the well-established epidemiological approach of estimating effects before and after adjustment and observing how much the effect size changes by the adjustment. Adjustment for the confounders on which measurements were available made little difference to the strength of associations seen, which is supportive but not proof of causality. If the occurrence of hypoglycaemia episodes makes it more likely for other events to be reported, then this could cause bias. This is unlikely to affect death ascertainment.

Several studies have shown that a high proportion of episodes of hypoglycaemia on continuous glucose monitoring (CGM) with glucose readings 3–3.9 and <3 mmol/l are asymptomatic [51]. Recent data from the InRange trial [52] showed that CGM captured 2–6-fold higher hypoglycaemia event rates than self-monitoring of blood glucose (SMBG) for both level 1 and level 2 hypoglycaemic episodes. A study has shown that people with type 1 diabetes typically spend 1.5 h per day with blood glucose levels below 3.9 mmol/l [53]. In the trials included here, level 1 and level 2 hypoglycaemia were ascertained mostly by symptomatic hypoglycaemia with a confirmatory blood glucose, with some events being asymptomatic hypoglycaemia detected by self-monitored blood glucose. Thus, many asymptomatic periods of hypoglycaemia may have been missed compared with what might be detected had CGM been available in the trials. Whether the capture of such asymptomatic events would increase or decrease the HRs for events associated with blood glucose in the hypoglycaemic range shown in Fig. 1 depends on whether the true HR for such events is higher or lower for asymptomatic vs symptomatic hypoglycaemia. The main implication is that the HRs shown in Fig. 1 should not be assumed to apply to asymptomatic hypoglycaemia. Confounding of associations by tendency to self-measure is possible if such a tendency is itself separately associated with the outcome.

Ascertainment would likely be stronger at lower blood glucose levels and so is less likely to explain associations with level 1 hypoglycaemia exposures. Our pre-specified 10 day interval used to explore association of adverse outcomes to recent hypoglycaemia exposure is arbitrary and a trade-off between capturing as many consequences as possible and keeping the time-frame reasonably acute. However, similarly with hypoglycaemia event ascertainment, our choice would reduce power rather than induce false associations. A substantial challenge was that the trials represented very different subpopulations, with prior SH within 12 months being an exclusion criterion in many of the trials (a subgroup who carry the most burden of hypoglycaemia and are therefore of the most interest). Exclusion criteria for unawareness of hypoglycaemia were trial dependent, with the effect of this being to underestimate the risk ratio associated with a given level of hypoglycaemia event. In most trials, participants who had multiple serious hypoglycaemia events over a 6 to 12 month period prior to a trial would not typically be included. Widely varying incidence rates of all levels of hypoglycaemia necessitated adjustment for study number in the analysis to avoid confounding by study entry criteria. With respect to our analysis, the data are observational and not randomised and we are not able to use trial randomisation as an instrument for causality.

### Conclusions

The data presented here reinforce the importance of hypoglycaemia for other clinical outcomes in diabetes. The data are consistent with worsening outcomes in a range of macro- and microvascular complications of diabetes with cumulative exposure to hypoglycaemia and provide supportive evidence for the use of level 2 and 3 hypoglycaemia, as recently approved by the EMA, as an endpoint in relevant trials. While for trial reporting and decision-making purposes it is useful to define different levels of hypoglycaemia, we did not find evidence to suggest that there is a threshold effect below a blood glucose of 3.9 mmol/l. This underscores the importance of preventing all hypoglycaemia episodes, not just for the obvious adverse impacts such episodes have on quality of life for people with diabetes, but also to prevent adverse clinical outcomes. Taking account of all hypoglycaemia may also help economic analyses of the potential wider benefits of hypoglycaemia prevention.

## Supplementary Information

Below is the link to the electronic supplementary material.

Supplementary file1 (PDF 267 KB)

## Data Availability

The data underlying the results presented in the study come from the Hypo-RESOLVE data repository. The repository will be maintained for a 2 year period following the end of the Hypo-RESOLVE project. Enquiries about third-party researcher data access and associated access criteria should be sent to the Hypo-RESOLVE data access committee (Chair: Jakob Haardt, J.Haardt@eurice.eu/Mark Ibberson, Mark.Ibberson@sib.swiss; or via https://hypo-resolve.eu/contact).

## Abbreviations

CGM: Continuous glucose monitoring
EMA: European Medicines Agency
FDA: Food and Drug Administration
Hypo-RESOLVE: Hypoglycaemia REdefining SOLutions for better liVEs
IHSG: International Hypoglycaemia Study Group
MedDRA: Medical Dictionary for Regulatory Activities
SH: Severe hypoglycaemia

## Authors’ relationships and activities

HMC has research grants from JDRF and Diabetes UK. She has participated in a speakers' bureau, on an advisory board for Novo Nordisk and on an advisory panel for Bayer AG, and is a shareholder of Roche Pharmaceuticals and Bayer AG. SH has participated on advisory boards for Eli Lilly and Novo Nordisk and in a speakers' bureau for Novo Nordisk, and received an honorarium via his institution from Medtronic. He has consulted for Vertex Pharma, Zucara Pharmaceuticals and Zealand Pharma. AS is an employee of, and holds shares in, Novo Nordisk. UP-B received a personal honorarium for lectures, presentations and for expert testimony from Sanofi and Novo Nordisk, and is a member of the editorial board of *Diabetologia*. BEdG is a member of the editorial board of *Diabetologia*. RJM received a personal honorarium for lectures and presentations from Sanofi and Novo Nordisk, and support for travel to lecture at an educational event from Sanofi. He is a non-executive member of NHS Tayside Health Board. MR is an employee of, and holds shares in, Eli Lilly and Company. SAA has served on advisory boards for Vertex Pharmaceuticals and spoken at educational events sponsored by Sanofi and Novo Nordisk. M-AG is an employee of, and holds shares in, Novo Nordisk. The authors declare that there are no other relationships or activities that might bias, or be perceived to bias, their work.
