# Supplementary material for: Estimating risk of consequences following hypoglycaemia exposure using the Hypo-RESOLVE cohort: a secondary analysis of pooled data from insulin clinical trials
Source: Diabetologia. 2024 Jul 22;67(10):2210–24. doi: 10.1007/s00125-024-06225-1 (PMC11447089; doi:10.1007/s00125-024-06225-1)
Supplement: Supplementary file 1 — Supplementary file1 (PDF 267 KB) [file 125_2024_6225_MOESM1_ESM.pdf]

ESM for Estimating risk of consequences following hypoglycaemia exposure using the Hypo-RESOLVE cohort: A secondary analysis of pooled data from insulin clinical trials

Joseph Mellor[1] Dmitry Kuznetsov[2] Simon Heller[12] Mari-Anne Gall[9]
Myriam Rosilio[10] Stephanie A. Amiel[11] Mark Ibberson[2]
Stuart McGurnaghan[3] Luke Blackbourn[3] William Berthon[1] Adel Salem[4]
Yongming Qu[5] Rory J. McCrimmon[6] Bastiaan E. de Galan[7]
Ulrik Pedersen-Bjergaard[8] Joanna Leaviss[13] Paul M. McKeigue[1]
Helen M. Colhoun[3]

Contents

ESM Methods **2**
CDISC domains and tables 2
Analysis of change in continuous outcomes 2

ESM Results **2**
Association of events with exposure with hypoglycaemia episodes in the previous 10 days 2
Analysis of change in continuous outcomes 2

References **2**

1. Usher Institute, College of Medicine and Veterinary Medicine, University of Edinburgh, Teviot Place, Edinburgh, EH8 9AG, UK
2. Swiss Institute of Bioinformatics, Lausanne, Switzerland
3. Institute of Genetics and Cancer, College of Medicine and Veterinary Medicine, University of Edinburgh, Western General Hospital Campus, Crewe Road, Edinburgh EH4 2XUC, UK
4. RW Data Assets, AI & Analytics (AIA), Novo Nordisk, Denmark
5. Eli Lilly and Company, Indianapolis, United States
6. Systems Medicine, School of Medicine, University of Dundee, Dundee, UK
7. Department of Internal Medicine, Division of Endocrinology and Metabolic Disease, Maastricht University Medical Center, Maastricht, the Netherlands
8. Institute of Clinical Medicine, University of Copenhagen, Copenhagen, Denmark
9. Medical & Science, Insulin, Clinical Drug Development, Novo Nordisk A/S, Denmark
10. Eli Lilly and Company, Diabetes Medical Unit, Neuilly sur seine, France
11. Department of Diabetes, School of Cardiovascular and Metabolic Medicine and Sciences, Faculty of Life Sciences and Medicine, King's College London, London, UK
12. Division of Clinical Medicine, University of Sheffield, Sheffield, UK
13. School of Health and Related Research (ScHARR), University of Sheffield, Sheffield, UK

ESM Methods

CDISC domains and tables

The data were organised in tables/domains including AE (adverse events), DM (demographics), EX (treatment exposure), VS (vital signs), SV (subject visits), MH (medical history), CM (concomitant medications), LB (laboratory measurements), TA (trial arms), SE (subject elements).

Analysis of change in continuous outcomes

We also evaluated the impact of hypoglycaemia on continuous measures. We conducted a simple analysis of the within-person change in the outcome following a hypoglycaemic episode for the following outcomes: HbA1c, systolic blood pressure, weight, eGFR, self-monitored blood glucose, and coefficient of variation of self-monitored blood glucose. For each hypoglycaemic episode we calculated the change in outcome between the most recent observation prior to the hypoglycaemic episode and earliest observation following the episode. Hypoglycaemic episodes which did not have a subsequent outcome observation, or did not have a prior outcome observation were dropped from the analysis. The distributions in change of outcome for each level of hypoglycaemia, for type 1 and type 2 diabetes separately, were simply plotted as histograms.

ESM Results

Association of events with exposure with hypoglycaemia episodes in the previous 10 days

As shown in ESM Table 6, in type 1 diabetes there was an increased risk of death, acute CVD and retinal disorders associated with recent hypoglycaemic episodes of any kind even with full adjustment for covariates. These associations varied in strength and significance in the less powerful analysis of each level of hypoglycaemia episode considered separately, shown in ESM Table 7.

For type 2 diabetes there was an increased risk of death, acute CVD and retinal disorders with recent hypoglycaemic episodes of any kind even with full covariate adjustment (see ESM Table 8). These associations were stronger than for type 1 diabetes and significant (see ESM Table 9) when level 1 and 2 episodes were considered separately.

Analysis of change in continuous outcomes

Pre- to post-hypoglyaemic episode change in continuous outcome

ESM Figures 1, 2, and 3 show the distribution of within person change in continuous outcomes before and after the occurrence of hypoglycaemic episodes for level 1, level 2, and level 3 episodes respectively. There was no evidence of any systematic change in either direction in systolic blood pressure, weight, eGFR, self-monitored blood glucose, or coefficient of variation based on self-monitored blood glucose in either type 1 or type 2 diabetes. This is shown by histograms being approximately symmetric about the origin. HbA1c measured following hypoglycaemic episodes was systematically lower than before such episodes, more substantially in type 2 diabetes.

References

ESM Table 1: MedDRA preferred term definitions for event outcomes used in analysis

Outcome	MEDDRAPT
CVD	Coronary artery disease, Myocardial ischaemia, Angina pectoris, Atrial fibrillation, Arteriosclerosis coronary artery, Cardiac failure congestive, Myocardial infarction, Cardiac failure, Cerebral infarction, Cardiac failure chronic, Cerebral ischaemia, Cerebrovascular accident, Angina unstable, Acute myocardial infarction, Left ventricular failure, Hemiparesis, Ischaemic stroke, Coronary angioplasty, Coronary arterial stent insertion, Coronary artery bypass, Hemiplegia, Acute coronary syndrome, Coronary artery occlusion, Pulmonary oedema, Cerebral artery occlusion, Monoparesis, Acute pulmonary oedema, Cardiac arrest, Cardio-respiratory arrest, Infarction, Coronary revascularisation, Cerebral haemorrhage, Ventricular fibrillation, Subarachnoid haemorrhage, Cardiac death, Haemorrhage intracranial, Cerebellar infarction, Intraventricular haemorrhage, Troponin increased, Right ventricular failure, Brain stem haemorrhage, Brain stem infarction, Acute left ventricular failure, Electrocardiogram Q wave abnormal, Cardiac failure acute, Silent myocardial infarction, Haemorrhagic stroke, Sudden cardiac death, Coronary artery dissection, Lacunar stroke, Ischaemic cerebral infarction
Retinal disorders	Diabetic retinopathy, Retinopathy, Retinal disorder, Macular oedema, Retinopathy hypertensive, Retinal haemorrhage, Retinopathy proliferative, Retinal vascular disorder, Maculopathy, Vitreous haemorrhage, Vitreous detachment, Eye haemorrhage, Blindness, Diabetic retinal oedema, Retinal laser coagulation
Neuropathy	Neuropathy peripheral, Polyneuropathy, Peripheral sensory neuropathy, Peripheral sensorimotor neuropathy, Peripheral motor neuropathy, Mononeuropathy
Kidney disease	Diabetic nephropathy, Microalbuminuria, Proteinuria, Nephropathy, Renal failure, Chronic kidney disease, Albuminuria, Urine albumin/creatinine ratio increased, Protein urine present, Albumin urine present, Creatinine renal clearance decreased, Urine protein/creatinine ratio increased, End stage renal disease
Depression	Depression, Major depression, Persistent depressive disorder, Suicidal ideation, Suicide attempt, Completed suicide, Depression suicidal, Suicidal behaviour

ESM Table 2: Medical History definitions

Condition	Description
Retinopathy	'Diabetic Retinopathy' MEDDRA preferred term in the MH table
Neuropathy	'Diabetic Neuropathy' MEDDRA preferred term in the MH table
Nephropathy	'Diabetic Nephropathy' MEDDRA preferred term in the MH table
CVD	Coronary artery disease, Myocardial ischaemia, Angina pectoris, Atrial fibrillation, Arteriosclerosis coronary artery, Cardiac failure congestive, Myocardial infarction, Cardiac failure, Cerebral infarction, Cardiac failure chronic, Cerebral ischaemia, Cerebrovascular accident, Angina unstable, Acute myocardial infarction, Left ventricular failure, Hemiparesis, Ischaemic stroke, Coronary angioplasty, Coronary arterial stent insertion, Coronary artery bypass, Hemiplegia, Acute coronary syndrome, Coronary artery occlusion, Pulmonary oedema, Cerebral artery occlusion, Monoparesis, Acute pulmonary oedema, Cardiac arrest, Cardio-respiratory arrest, Infarction, Coronary revascularisation, Cerebral haemorrhage, Ventricular fibrillation, Subarachnoid haemorrhage, Cardiac death, Haemorrhage intracranial, Cerebellar infarction, Intraventricular haemorrhage, Troponin increased, Right ventricular failure, Brain stem haemorrhage, Brain stem infarction, Acute left ventricular failure, Electrocardiogram Q wave abnormal, Cardiac failure acute, Silent myocardial infarction, Haemorrhagic stroke, Sudden cardiac death, Coronary artery dissection, Lacunar stroke, Ischaemic cerebral infarction

ESM Table 3: Concomitant Medications definitions

Concomitant Medication	Description
Anti-epileptics	ATC level 2 codes for anti-epileptics (N03)
Psychoactive agents	ATC level 3 codes for Antidepressants (N06A), and Opioids (N02A). ATC level 4 codes for Benzodiazepine derivatives (N05BA), and Antidepressants in combination with psycholeptics (N06CA). ATC level 5 codes for Cannabinoids (includes nabiximols) (N02BG10), Cocaine (N01BC01), and Haloperidol (N05AD01)
Blood-glucose lowering drugs	Blood-glucose lowering drugs excluding insulin ATC level 3 code A10B
Systemic Oral Anti-inflammatories	ATC level 2 codes for Anti-inflammatory and antirheumatic products (M01), ATC level 3 codes for Intestinal anti-inflammatory agents (A07E) and Other analgesics and antipyretics (N02B), and ATC level 4 codes for Anti-inflammatory products for vaginal administration (G02CC)
Anti-hypertensives	ATC level 2 codes for: Anti-hypertensives (C02), Diuretics (C03), Beta blocking agents (C07), Calcium channel blockers (C08), and agents acting on the renin-angiotensin system

ESM Table 4: Adjustment covariates in long-term cumulative exposure to hypoglycaemia fully-adjusted models.

Adverse event	T1Covariates	T2Covariates
Death	Study Identifier, Age, Diabetes duration, HbA_{1c}, Sex, Insulin origin, Total daily insulin dose, Ethnicity, Self-monitored blood glucose, Self-monitored blood glucose coefficient of variation, CVD at baseline, Retinopathy at baseline, Neuropathy at baseline, Nephropathy at baseline, Antihypertensives, Antiinflammatories, Psychoactives, Anti-epileptics, Blood glucose lowering drugs	Study Identifier, Age, Diabetes duration, HbA_{1c}, Sex, Insulin regimen, Insulin origin, Total daily insulin dose, Ethnicity, Self-monitored blood glucose, Self-monitored blood glucose coefficient of variation, CVD at baseline, Retinopathy at baseline, Neuropathy at baseline, Nephropathy at baseline, Antihypertensives, Antiinflammatories, Psychoactives, Anti-epileptics, Blood glucose lowering drugs
CVD	Study Identifier, Age, Diabetes duration, HbA_{1c}, Sex, Insulin origin, Total daily insulin dose, Ethnicity, Self-monitored blood glucose, Self-monitored blood glucose coefficient of variation, CVD at baseline, Retinopathy at baseline, Neuropathy at baseline, Nephropathy at baseline, Antihypertensives, Antiinflammatories, Psychoactives, Anti-epileptics, Blood glucose lowering drugs	Study Identifier, Age, Diabetes duration, HbA_{1c}, Sex, Insulin regimen, Insulin origin, Total daily insulin dose, Ethnicity, Self-monitored blood glucose, Self-monitored blood glucose coefficient of variation, CVD at baseline, Retinopathy at baseline, Neuropathy at baseline, Nephropathy at baseline, Antihypertensives, Antiinflammatories, Psychoactives, Anti-epileptics, Blood glucose lowering drugs
Neuropathy	Study Identifier, Age, Diabetes duration, HbA_{1c}, Sex, Insulin origin, Total daily insulin dose, Ethnicity, Self-monitored blood glucose, Self-monitored blood glucose coefficient of variation, CVD at baseline, Retinopathy at baseline, Neuropathy at baseline, Nephropathy at baseline, Antihypertensives, Antiinflammatories, Psychoactives, Anti-epileptics, Blood glucose lowering drugs	Study Identifier, Age, Diabetes duration, HbA_{1c}, Sex, Insulin regimen, Insulin origin, Total daily insulin dose, Ethnicity, Self-monitored blood glucose, Self-monitored blood glucose coefficient of variation, CVD at baseline, Retinopathy at baseline, Neuropathy at baseline, Nephropathy at baseline, Antihypertensives, Antiinflammatories, Psychoactives, Anti-epileptics, Blood glucose lowering drugs
Kidney disease	Study Identifier, Age, Diabetes duration, HbA_{1c}, Sex, Insulin origin, Total daily insulin dose, Ethnicity, Self-monitored blood glucose, Self-monitored blood glucose coefficient of variation, CVD at baseline, Retinopathy at baseline, Neuropathy at baseline, Nephropathy at baseline, Antihypertensives, Antiinflammatories, Psychoactives, Anti-epileptics, Blood glucose lowering drugs	Study Identifier, Age, Diabetes duration, HbA_{1c}, Sex, Insulin regimen, Insulin origin, Total daily insulin dose, Ethnicity, Self-monitored blood glucose, Self-monitored blood glucose coefficient of variation, CVD at baseline, Retinopathy at baseline, Neuropathy at baseline, Nephropathy at baseline, Antihypertensives, Antiinflammatories, Psychoactives, Anti-epileptics, Blood glucose lowering drugs
Retinal disorders	Study Identifier, Age, Diabetes duration, HbA_{1c}, Sex, Insulin origin, Total daily insulin dose, Ethnicity, Self-monitored blood glucose, Self-monitored blood glucose coefficient of variation, CVD at baseline, Retinopathy at baseline, Neuropathy at baseline, Nephropathy at baseline, Antihypertensives, Antiinflammatories, Psychoactives, Anti-epileptics, Blood glucose lowering drugs	Study Identifier, Age, Diabetes duration, HbA_{1c}, Sex, Insulin regimen, Insulin origin, Total daily insulin dose, Ethnicity, Self-monitored blood glucose, Self-monitored blood glucose coefficient of variation, CVD at baseline, Retinopathy at baseline, Neuropathy at baseline, Nephropathy at baseline, Antihypertensives, Antiinflammatories, Psychoactives, Anti-epileptics, Blood glucose lowering drugs
Depression	Study Identifier, Age, Diabetes duration, HbA_{1c}, Sex, Insulin origin, Total daily insulin dose, Ethnicity, Self-monitored blood glucose, Self-monitored blood glucose coefficient of variation, CVD at baseline, Retinopathy at baseline, Neuropathy at baseline, Nephropathy at baseline, Antihypertensives, Antiinflammatories, Psychoactives, Anti-epileptics, Blood glucose lowering drugs	Study Identifier, Age, Diabetes duration, HbA_{1c}, Sex, Insulin regimen, Insulin origin, Total daily insulin dose, Ethnicity, Self-monitored blood glucose, Self-monitored blood glucose coefficient of variation, CVD at baseline, Retinopathy at baseline, Neuropathy at baseline, Nephropathy at baseline, Antihypertensives, Antiinflammatories, Psychoactives, Anti-epileptics, Blood glucose lowering drugs

ESM Table 5: Adjustment covariates in recent exposure to hypoglycaemia fully-adjusted models.

Adverse event	T1Covariates	T2Covariates
Death	Study Identifier, Age, Diabetes duration, HbA_{1c}, Sex, Insulin origin, Total daily insulin dose, Ethnicity, Self-monitored blood glucose, Self-monitored blood glucose coefficient of variation, CVD at baseline, Retinopathy at baseline, Neuropathy at baseline, Nephropathy at baseline, Antihypertensives, Antiinflammatories, Psychoactives, Anti-epileptics, Blood glucose lowering drugs	Study Identifier, Age, Diabetes duration, HbA_{1c}, Sex, Insulin regimen, Insulin origin, Total daily insulin dose, Ethnicity, Self-monitored blood glucose, Self-monitored blood glucose coefficient of variation, CVD at baseline, Retinopathy at baseline, Neuropathy at baseline, Nephropathy at baseline, Antihypertensives, Antiinflammatories, Psychoactives, Anti-epileptics, Blood glucose lowering drugs
CVD	Study Identifier, Age, Diabetes duration, HbA_{1c}, Sex, Insulin origin, Total daily insulin dose, Ethnicity, Self-monitored blood glucose, Self-monitored blood glucose coefficient of variation, CVD at baseline, Retinopathy at baseline, Neuropathy at baseline, Nephropathy at baseline, Antihypertensives, Antiinflammatories, Psychoactives, Anti-epileptics, Blood glucose lowering drugs	Study Identifier, Age, Diabetes duration, HbA_{1c}, Sex, Insulin regimen, Insulin origin, Total daily insulin dose, Ethnicity, Self-monitored blood glucose, Self-monitored blood glucose coefficient of variation, CVD at baseline, Retinopathy at baseline, Neuropathy at baseline, Nephropathy at baseline, Antihypertensives, Antiinflammatories, Psychoactives, Anti-epileptics, Blood glucose lowering drugs
Retinal disorders	Study Identifier, Age, Diabetes duration, HbA_{1c}, Sex, Insulin origin, Total daily insulin dose, Ethnicity, Self-monitored blood glucose, Self-monitored blood glucose coefficient of variation, CVD at baseline, Retinopathy at baseline, Neuropathy at baseline, Nephropathy at baseline, Antihypertensives, Antiinflammatories, Psychoactives, Anti-epileptics, Blood glucose lowering drugs	Study Identifier, Age, Diabetes duration, HbA_{1c}, Sex, Insulin regimen, Insulin origin, Total daily insulin dose, Ethnicity, Self-monitored blood glucose, Self-monitored blood glucose coefficient of variation, CVD at baseline, Retinopathy at baseline, Neuropathy at baseline, Nephropathy at baseline, Antihypertensives, Antiinflammatories, Psychoactives, Anti-epileptics, Blood glucose lowering drugs

ESM Table 6: Rate ratios giving the increase in rate of outcome event for every standard deviation increase in log(1 + 10-day exposure to all hypoglycaemia) for various outcomes for type 1 diabetes.

			Hypogycaemia of any level	
Adverse event	Individuals	Events	Total Exposure	Rate ratio
**Minimally-adjusted**				
Death	4901	21	474031	**1.341 (1.04, 1.729)**
CVD	9486	90	787600	**1.527 (1.245, 1.874)**
Retinal disorders	9744	328	797095	**1.72 (1.59, 1.861)**
**Fully-adjusted**				
Death	4765	21	466710	**1.315 (1.05, 1.648)**
CVD	6823	73	671674	**1.551 (1.224, 1.964)**
Retinal disorders	6665	240	657094	**1.65 (1.5, 1.814)**

Note:

Rate ratios are given with 95% confidence intervals

Bold type indicates statistically significant rate ratios

Definitions of adverse events in terms of MedDRA preferred terms can be found in this document

Adjustment covariates for fully-adjusted models can be found in this document

ESM Table 7: Rate ratios giving the increase in rate of outcome event for every standard deviation increase in log(1 + 10-day exposure to hypoglycaemia) for various outcomes for type 1 diabetes.

			Level 1	Level 2	Level 3
Adverse event	Individuals	Events	Rate ratio	Rate ratio	Rate ratio
**Minimally-adjusted**					
Death	4901	21	1.071 (0.804, 1.427)	1.376 (0.914, 2.071)	1.032 (0.843, 1.265)
CVD	9486	90	1.134 (0.941, 1.368)	**1.499 (1.171, 1.919)**	**1.133 (1.051, 1.222)**
Retinal disorders	9744	328	**1.334 (1.195, 1.488)**	**1.456 (1.322, 1.603)**	0.995 (0.938, 1.056)
**Fully-adjusted**					
Death	4765	21	1.075 (0.845, 1.367)	1.344 (0.91, 1.985)	1.014 (0.814, 1.264)
CVD	6823	73	1.209 (0.987, 1.48)	**1.45 (1.12, 1.878)**	**1.121 (1.042, 1.206)**
Retinal disorders	6665	240	**1.251 (1.108, 1.414)**	**1.443 (1.29, 1.613)**	0.993 (0.933, 1.057)

Note:
Rate ratios are given with 95% confidence intervals
Bold type indicates statistically significant rate ratios
Definitions of adverse events in terms of MedDRA preferred terms can be found in supplementary material
Adjustment covariates for fully-adjusted models can be found in supplementary material

ESM Table 8: Rate ratios giving the increase in rate of outcome event for every standard deviation increase in log(1 + 10-day exposure to all hypoglycaemia) for various outcomes for type 2 diabetes.

			Hypogycaemia of any level	
Adverse event	Individuals	Events	Total Exposure	Rate ratio
**Minimally-adjusted**				
Death	15408	92	202600	**2.497 (2.149, 2.901)**
CVD	27009	719	296126	**2.701 (2.495, 2.924)**
Retinal disorders	26706	679	292838	**2.578 (2.393, 2.776)**
**Fully-adjusted**				
Death	14195	89	199757	**2.415 (2.003, 2.911)**
CVD	20662	584	272442	**2.681 (2.476, 2.903)**
Retinal disorders	20481	585	271880	**2.471 (2.303, 2.651)**

Note:
Rate ratios are given with 95% confidence intervals
Bold type indicates statistically significant rate ratios
Definitions of adverse events in terms of MedDRA preferred terms can be found in supplementary material
Adjustment covariates for fully-adjusted models can be found in supplementary material

ESM Table 9: Rate ratios giving the increase in rate of outcome event for every standard deviation increase in log(1 + 10-day exposure to hypoglycaemia) for various outcomes for type 2 diabetes.

			Level 1	Level 2	Level 3
Adverse event	Individuals	Events	Rate ratio	Rate ratio	Rate ratio
**Minimally-adjusted**					
Death	15408	92	**1.873 (1.511, 2.321)**	**1.531 (1.182, 1.981)**	1.093 (0.956, 1.25)
CVD	27009	719	**2.091 (1.936, 2.26)**	**1.44 (1.301, 1.594)**	**1.14 (1.063, 1.222)**
Retinal disorders	26706	679	**2.289 (2.131, 2.46)**	**1.252 (1.155, 1.356)**	1.056 (0.969, 1.151)
**Fully-adjusted**					
Death	14195	89	**1.862 (1.51, 2.297)**	**1.465 (1.111, 1.932)**	1.094 (0.954, 1.255)
CVD	20662	584	**2.107 (1.952, 2.274)**	**1.412 (1.291, 1.544)**	**1.145 (1.081, 1.212)**
Retinal disorders	20481	585	**2.248 (2.099, 2.408)**	**1.196 (1.102, 1.298)**	1.047 (0.953, 1.15)

Note:
Rate ratios are given with 95% confidence intervals
Bold type indicates statistically significant rate ratios
Definitions of adverse events in terms of MedDRA preferred terms can be found in supplementary material
Adjustment covariates for fully-adjusted models can be found in supplementary material

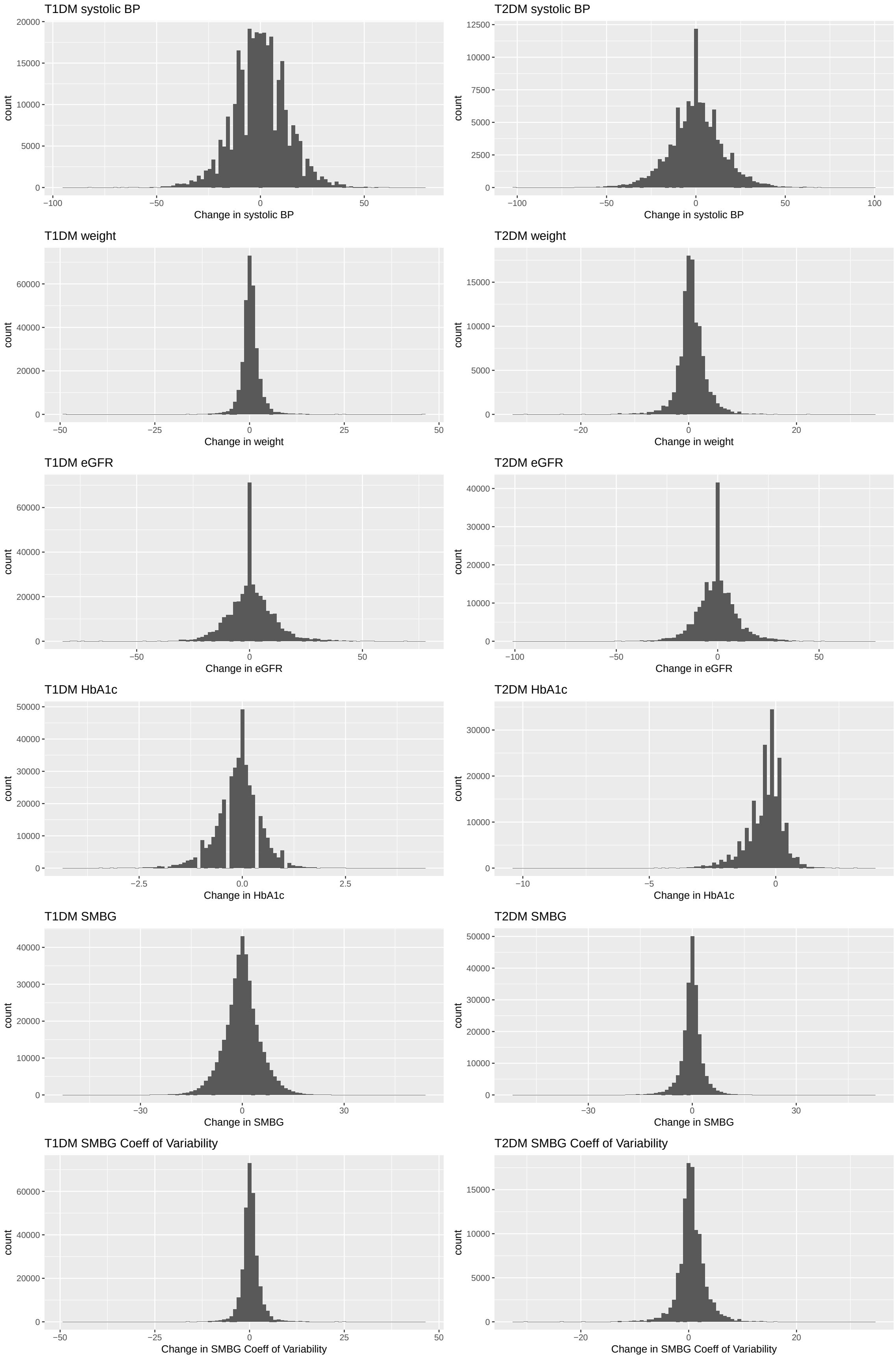

ESM Figure 1: Histograms of change in outcome pre- and post- level 1 hypoglycaemic episodes.

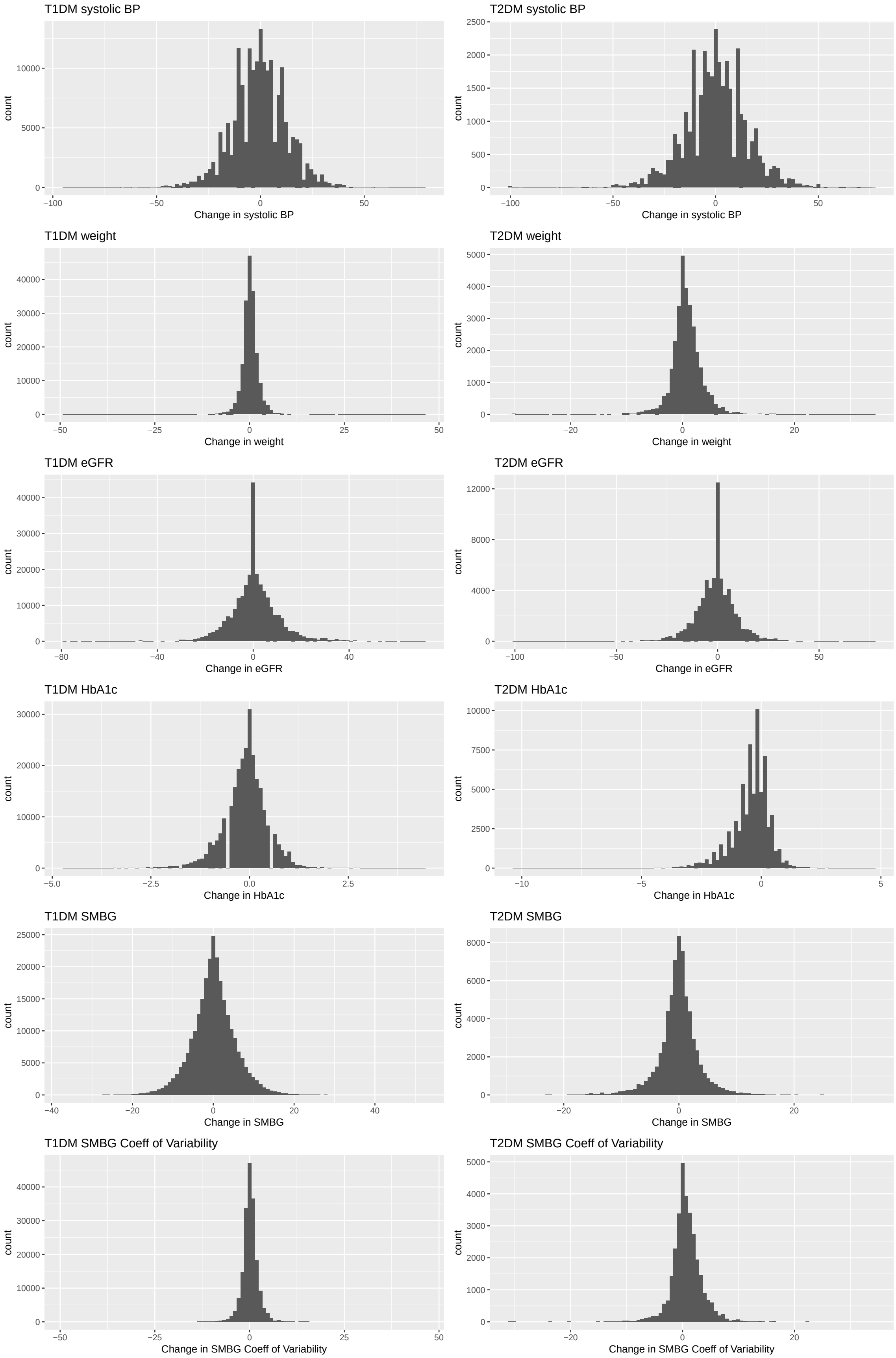

ESM Figure 2: Histograms of change in outcome pre- and post- level 2 hypoglycaemic episodes.

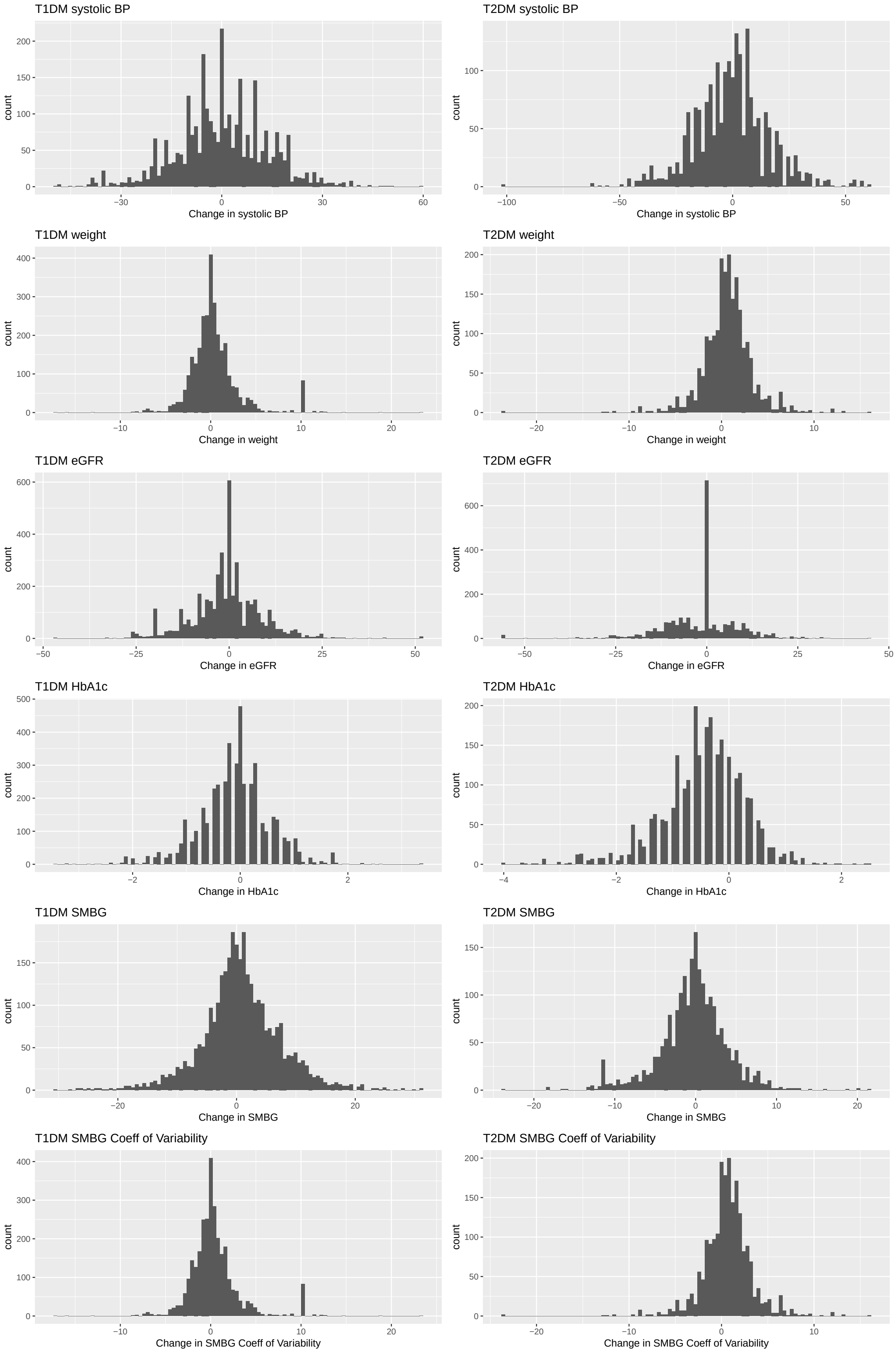

ESM Figure 3: Histograms of change in outcome pre- and post- level 3 hypoglycaemic episodes.
